# NNMT promotes the progression of intrahepatic cholangiocarcinoma by regulating aerobic glycolysis via the EGFR-STAT3 axis

**DOI:** 10.1038/s41389-022-00415-5

**Published:** 2022-07-18

**Authors:** Shounan Lu, Shanjia Ke, Chaoqun Wang, Yanan Xu, Zihao Li, Keda Song, Miaoyu Bai, Menghua Zhou, Hongjun Yu, Bing Yin, Xinglong Li, Zhigang Feng, Yongliang Hua, Shangha Pan, Hongchi Jiang, Linqiang Li, Yaohua Wu, Yong Ma

**Affiliations:** 1 Department of Minimal Invasive Hepatic Surgery, The First Affiliated Hospital of Harbin Medical University, Harbin, China; 2 Key Laboratory of Hepatosplenic Surgery, Ministry of Education, The First Affiliated Hospital of Harbin Medical University, Harbin, China; 3 Department of Hepatic Surgery, The First Affiliated Hospital of Harbin Medical University, Harbin, China; 4 Department of General Surgery, Linyi Central Hospital, Linyi, China; 5 The First Department of General Surgery, Affiliated Hospital of Inner Mongolia Minzu University, Tongliao, China; 6 Department of Pediatric Surgery, The First Affiliated Hospital of Harbin Medical University, Harbin, China; 7 Department of Thyroid Surgery, The First Affiliated Hospital of Harbin Medical University, Harbin, China

**Keywords:** Liver cancer, Epigenetics

## Abstract

Nicotinamide N-methyltransferase (NNMT), a member of the N-methyltransferase family, plays an important role in tumorigenesis. However, its expression and biological functions in intrahepatic cholangiocarcinoma (iCCA) remain to be established. In our study, we identified NNMT as an oncogene in iCCA and provided mechanistic insights into the roles of NNMT in iCCA progression. High NNMT expression in iCCA tissues was identified using western blotting and immunohistochemistry (IHC). We identified a significantly higher NNMT expression level in human iCCA tissues than that in adjacent normal tissues. Increased NNMT expression promoted iCCA cell proliferation and metastasis in vitro and in vivo. Mechanistically, NNMT inhibited the level of histone methylation in iCCA cells by consuming the methyl donor S-adenosyl methionine (SAM), thereby promoting the expression of epidermal growth factor receptor (EGFR). EGFR may activate the aerobic glycolysis pathway in iCCA cells by activating the STAT3 signaling pathway. In conclusion, we identified NNMT as an oncogene in iCCA and provided mechanistic insights into the roles of NNMT in iCCA progression.

## Introduction

Intrahepatic cholangiocarcinoma (iCCA) is a malignant tumor caused by abnormal bile duct epithelial cells [1]. Due to the lack of suitable diagnostic biomarkers, most patients with iCCA are diagnosed with advanced disease stage [2–4]. The possibility of improving the efficacy of iCCA treatment depends on an understanding of its molecular pathogenesis and the development of reasonable treatment methods to interfere with the oncogene signaling network driving and maintaining the development of iCCA. Despite an increasing number of clinical researches and early application of targeted drugs, we still lack a detailed understanding of the complex mechanisms that lead to the development and treatment of iCCA. Obviously, multiply treatment options are significant to patients with advanced and recurrent.

Nicotinamide N-methyltransferase (NNMT) is a cytosolic enzyme that belongs to the N-methyltransferase family [5]. NNMT consumes the general SAM to catalyze the methylation of nicotinamide (NAM) and structure compounds with related structures to generate S-adenosyl-L-homocysteine (SAH) and N1-methylnicotinamide (MNAM) [6]. NNMT is involved in the regulation of various metabolic pathways in tissues, such as adipose tissue and liver by affecting the methylation process and producing active metabolites [7]. Nicotinamide, a product of vitamin B3 and a precursor of NAD+, is essential to regulate energy metabolism and affects cell lifespan [8]. An increase in NNMT activity decreases nicotinamide levels and effectively inhibits apoptosis [9]. In healthy tissues, NNMT is mainly expressed in the liver, but low expression has been detected in the brain, heart, kidney, and so on [6, 10].

Epidermal growth factor receptor (EGFR) is generally upregulated in various cancer types and plays an important role in promoting cancer growth and metastasis [11]. However, many clinical trials have failed to show that the survival rate of patients with CCA who are treated with gefitinib or erlotinib is significantly improved [12, 13]. These data underscore the importance of better understanding the underlying intracellular mechanisms of EGFR resistance, as EGFR signaling affects CCA progression.

Researchers have found significantly higher NNMT expression in a variety of tumor cells. The upregulation of NNMT may be related to the proliferation of various cancer cells, including esophageal squamous cell carcinoma [14], prostate cancer [15], neuroblastoma [16], lung cancer [17], gastric cancer [18], ovarian cancer [19], and breast cancer [20]. In addition, the high expression of NNMT in these tumors positively correlates with tumor size and progression, suggesting that NNMT may regulate the initial stage of malignant transformation. However, the potential mechanism of NNMT in iCCA is unclear. In the present study, we found that NNMT promotes the proliferation and metastasis of iCCA cells by activating the EGFR-STAT3 signaling pathway.

## Methods

### Human tissue

Between 2010 and 2019, 80 iCCA and nontumor adjacent tissue specimens were collected at the First Affiliated Hospital of Harbin Medical University. Ethical approval was acquired from the Research Ethics Committee of the First Affiliated Hospital of Harbin Medical University, and each patient provided informed consent. The detailed clinicopathological characteristics of all patients with iCCA are listed in Supplementary Table 1.

### ATP concentrations, glucose consumption, and lactate secretion

Adenosine triphosphate (ATP) concentrations, glucose consumption, and lactate secretion were measured as described in our previous report [21]. ATP concentrations were quantified with an ATP Determination Kit (Beyotime, China) using a VarioSkan flash fluorescence plate reader (Thermo Scientific, USA) according to the manufacturer’s instructions. Glucose levels in the culture medium were measured using an assay kit from Nanjing Jiancheng Bioengineering Institute (Nanjing, China). The lactate level in the culture medium was detected using a lactate assay kit (Biovision Inc., USA). All data were normalized by cell number.

### ECAR and OCR

Oxygen consumption rate (OCR) and extracellular acidification rate (ECAR) in iCCA cells were measured using a Seahorse XF96 flux analyzer (Seahorse Bioscience, Billerica, Massachusetts, USA) according to the manufacturer’s instructions. Data were normalized to total protein content.

### ChIP and PCR amplification

Formaldehyde fixation, cell lysis, and sonication were conducted as previously described [22]. In total, 1 μg anti-H3K9me3, 1 μg anti-H3K27me3, or 1 μg of nonspecific immunoglobulin G (Santa Cruz) was used to chromatin immunoprecipitate. Input and immunoprecipitated DNA were subjected to reversal of the cross-links and purification followed by real-time PCR experiments. A complete list of primer sets is provided in Supplementary Table 2. The product was run on a 5% polyacrylamide gel. Electrophoresis results were quantified using PhosphoImager (Molecular Dynamics) and Image Quant software.

### Statistical analysis

Statistical analyses were performed using GraphPad Prism 9 software. Student’s *t*-test or one-way ANOVA was applied to determine the significance of differences between groups. Overall survival was estimated using the Kaplan–Meier method, and significance was determined using the log-rank test. The statistical correlation between the clinical parameters of patients with iCCA and different NNMT expression levels in Supplementary Table 1 was analyzed using the chi-square test or Fisher’s exact chi-square test. The correlation between NNMT and EGFR expression was analyzed by calculating the Pearson correlation coefficient. Statistical significance was determined at *P < 0.05, **P < 0.01, ***P < 0.001; n.s. represents not statistically significant.

More detailed methods are provided in the Supplementary Material.

## Results

### NNMT is expressed at high levels in human iCCA tissues and cell lines

**Fig. 1:**
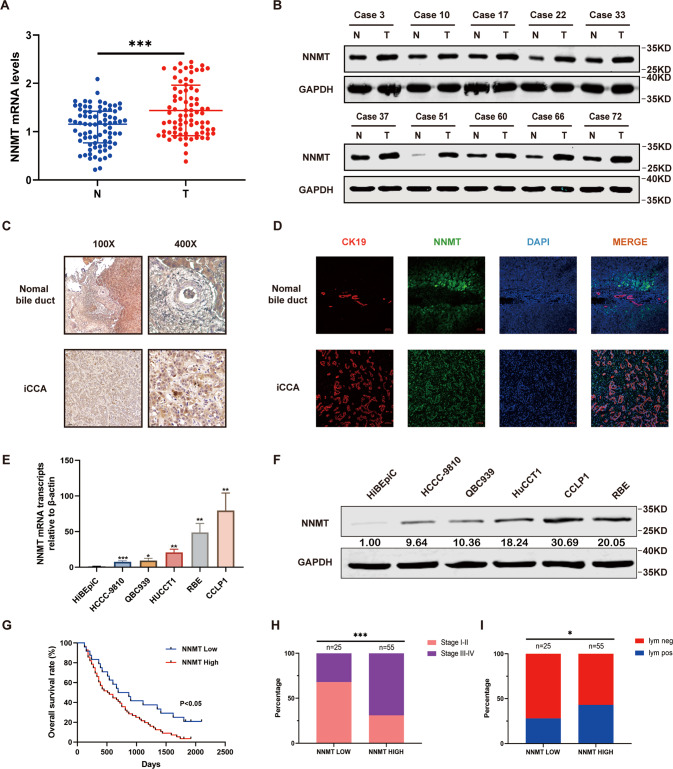
NNMT is expressed at high levels in iCCA tissues and cell lines. **A**, **B** The expression levels of the NNMT mRNA (**A**) and protein (**B**) in iCCA and adjacent tissues. **C** Representative images of NNMT IHC staining in iCCA tissues and adjacent tissues. Scale bars, ×100: 100 μm; ×400: 20 μm. **D** Representative images of NNMT IF staining in iCCA tissues and adjacent nomal bile duct tissues. Scale bars, 50 μm. **E**, **F** The expression levels of the NNMT mRNA (**D**) and protein (**E**) in various iCCA cell lines and normal bile duct cell line. **G** Kaplan–Meier curves of patients segregated by low/negative or high expression of NNMT. **H** Percentage of patients with AJCC stage I–IV iCCA segregated by low or high expression of NNMT. **I** Percentage of patients with lymph node metastases segregated by low or high expression of NNMT (data are mean ± SEM, *P < 0.05, **P < 0.01, ***P < 0.001, *n* = 3).

The qRT–PCR results showed higher expression of the NNMT mRNA in iCCA tumor tissues than that in adjacent tissues (Fig. 1A). Similarly, NNMT was more expressed in tumor tissues from 10 pairs of iCCA specimens than in paracancerous tissues (Fig. 1B). The results from IHC staining showed a higher NNMT staining score in iCCA than that in adjacent tissues (Fig. 1C). The IF experiment results also showed that the expression of NNMT in cholangiocarcinoma cells was significantly higher than that in adjacent normal bile duct cells (Fig. 1D). The NNMT mRNA and protein were expressed at higher levels in iCCA cell lines than in the normal bile duct epithelial cell line HiBEpiC (Fig. 1E, F). According to the IHC results, patients with iCCA were divided into two groups: the group with high NNMT expression and the group with low NNMT expression. Surprisingly, a significant correlation between high NNMT expression and shorter overall survival was observed (Fig. 1G). Moreover, patients with relatively high NNMT expression in tumors were significantly more likely to have advanced tumors and metastatic diseases (Fig. 1H, I). Based on these results, relatively high NNMT expression is positively correlated with a poor prognosis for patients with iCCA.

### NNMT promotes the proliferation and metastasis of iCCA in vitro and in vivo

**Fig. 2:**
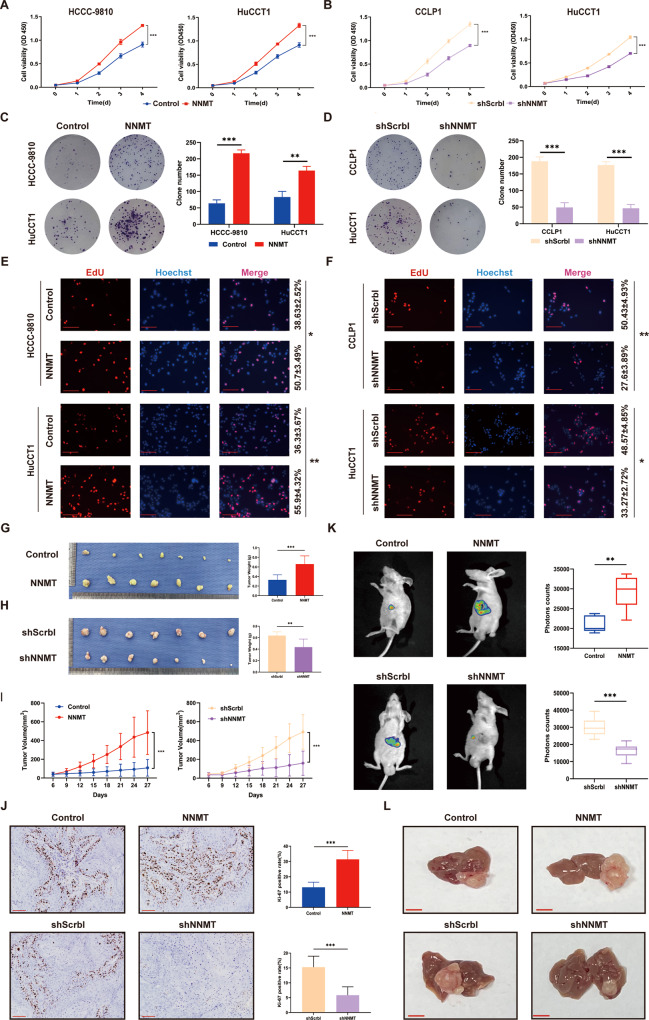
NNMT promotes the proliferation of iCCA cells in vitro and in vivo. **A**, **B** The CCK-8 experiment was used to evaluate the proliferation of NNMT-overexpressing (**A**) or -knockdown cells (**B**) and the control iCCA cell line (*n* = 3). **C**, **D** Representative image of the colony formation experiment evaluating the proliferation of NNMT-overexpressing (**C**) or -knockdown cells (**D**) and the control iCCA cell line (*n* = 3). **E**, **F** Representative image of the EdU proliferation experiment evaluating the proliferation of NNMT-overexpressing (**E**) or -knockdown cells (**F**) and the control iCCA cell line. Scale bars: 100 μm (*n* = 3). **G**, **H** Images of subcutaneous xenografts derived from the indicated cell lines and tumor weight of subcutaneous tumors produced from the indicated cell lines. Seven mice were included in each group (*n* = 7). **I** Tumor growth curves of subcutaneous xenografts. The tumor volume was measured every 3 days starting on the 6th day (*n* = 7). **J** Representative images of IHC staining for Ki-67 in subcutaneous xenograft models derived from the indicated cell lines. Scale bars: 100 μm (*n* = 7). **K** Representative images of livers from liver xenograft models derived from the indicated cell lines (*n* = 7). **L** Representative images of liver xenografts derived from the indicated cell lines. Scale bars: 1 cm (data are mean ± SEM, *P < 0.05, **P < 0.01, ***P < 0.001).

First, we overexpressed NNMT in normal bile duct epithelial cells HiBEpiC, and then performed CCK-8 experiments and transwell experiments. The results showed that NNMT had no significant effect on the proliferation and metastasis of HiBEpiC (Supplementary Fig. 1). Next, we assessed the role of NNMT in iCCA by transfecting NNMT overexpression lentivirus into HCCC-9810 and HuCCT1 cell lines and transfecting lentivirus into CCLP1 and HuCCT1 cell lines to silence NNMT. We conducted CCK-8 assays to evaluate the effect of NNMT on the proliferation of iCCA cells. NNMT overexpression significantly increased the proliferation of iCCA cells compared to the control group, while knockdown of NNMT inhibited the proliferation of iCCA cells (Fig. 2A, B). Colony formation assays showed that cells overexpressing NNMT formed more colonies, while cells in which NNMT was silenced produced fewer colonies than the control (Fig. 2C, D). The EdU experiment also indicated that NNMT induced the proliferation of iCCA cells (Fig. 2E, F).

In vivo, NNMT overexpression promoted tumorigenicity to a certain extent, while NNMT knockdown resulted in a smaller tumor volume and tumor weight (Fig. 2G–I). IHC staining indicated that Ki-67 expression was obviously decreased in NNMT-knockdown xenograft tumors and increased in NNMT-overexpressing xenograft tumors (Fig. 2J). We further determined whether NNMT regulates the malignant progression of iCCA in vivo, by generating an orthotopic xenograft model in which control and NNMT overexpression or NNMT knockdown iCCA cells were inoculated into the livers of nude mice. As expected, NNMT significantly regulated the growth of iCCA tumors (Fig. 2K, L).

**Fig. 3:**
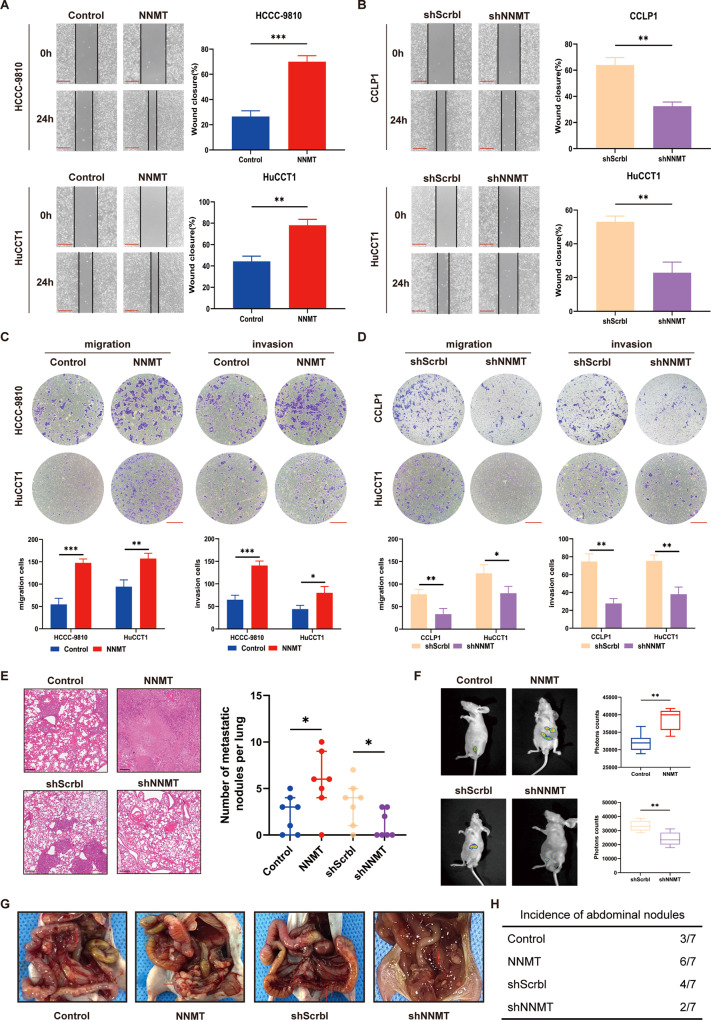
NNMT promotes the migration and invasion of iCCA cells in vitro and in vivo. **A**, **B** NNMT-overexpressing (**A**), -knockdown (**B**), and control cell lines were used in wound-healing experiments. Scale bars: 100 μm (*n* = 3). **C**, **D** Transwell experiments verified the migration and invasion of cells overexpressing (**C**) or knockdown (**D**) NNMT, as well as the control cell lines. Scale bars: 100 μm (*n* = 3). **E** Representative images of H&E staining in lung metastases (left panel) and the number of metastatic nodules per lung (right panel) (*n* = 7). **F**, **G** Representative images of the tumor nodules in peritoneal metastasis experiments (*n* = 7). **H** The number of nude mice with metastatic nodules in each group (data are mean ± SEM, *P < 0.05, **P < 0.01, ***P < 0.001).

In addition to the effect on proliferation, NNMT also modulates cell migration and invasion. Wound-healing assays showed that NNMT promoted the migration of iCCA cells (Fig. 3A, B). Matrigel-uncoated and matrigel-coated transwell assays revealed the increased migration and invasion of iCCA cells overexpressing NNMT, whereas the migration and invasion of iCCA cells were weakened after NNMT knockdown (Fig. 3C, D). Next, we injected iCCA cells into the tail vein or abdominal cavity of nude mice and observed the effect of NNMT on metastasis. NNMT knockdown reduced the size of iCCA lung metastases, and fewer metastatic nodules were observed in the abdominal cavity compared to the control group (Fig. 3E–H). In summary, NNMT promotes the proliferation, migration, and invasion of iCCA cells in vivo and in vitro.

### NNMT promotes the Warburg effect in iCCA

**Fig. 4:**
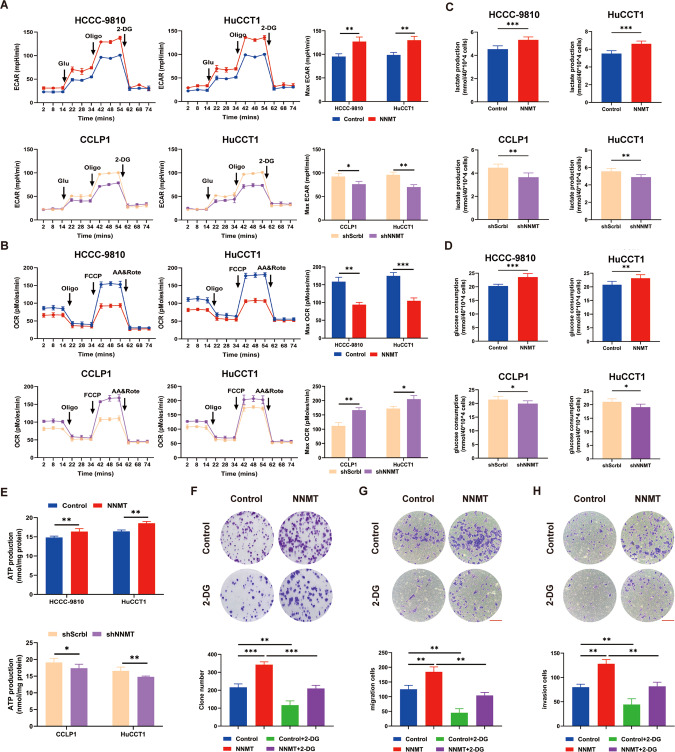
NNMT promotes the progression of iCCA by regulating the Warburg effect. **A** The Seahorse X96 analyzer detects the ability of iCCA cell lines to excrete acid. **B** Seahorse X96 detects the oxygen consumption capacity of iCCA cell lines. Glu, glucose; Oligo, oligomycin; 2-DG, 2-deoxyglucose; FCCP, carbonyl cyanide 4-[trifluoromethoxy] phenylhydrazone; AA&Rote, antimycin A and rotenone. **C** The amount of lactic acid produced in iCCA cell lines. **D** Glucose consumption was measured in iCCA cell lines. **E** The amount of ATP produced in iCCA cell lines. **F** The colony formation experiment detected cell growth after the addition of 4 μM 2-DG to the culture medium. **G** The Transwell experiment detected cell migration after the addition of 4 μM 2-DG to the culture medium. **H** The Transwell experiment detected cell invasion after the addition to 4 μM 2-DG to the culture medium. Scale bars: 100 μm (data are mean ± SEM, *P < 0.05, **P < 0.01, ***P < 0.001, *n* = 3).

Many studies have confirmed that aerobic glycolysis plays an important role in tumor cells. NNMT has been confirmed to be involved in methyl metabolism, lipid metabolism and glucose metabolism in cells. Therefore, we speculated that NNMT may participate in the aerobic glycolysis in iCCA cells to promote proliferation and metastasis. We confirmed our hypothesis by comparing the key cell metabolism and bioenergy parameters of iCCA cell lines with NNMT overexpression or knockdown and control cell lines. NNMT overexpression significantly decreased basal and maximal OCRs and increased ECARs in HCCC-9810 and HuCCT1 cells (Fig. 4A, B). In contrast, NNMT knockdown increased the OCR and decreased the ECAR in CCLP1 and HuCCT1 cells (Fig. 4A, B) In addition, overexpression of NNMT resulted in increased intracellular ATP levels, glucose uptake, and extracellular lactate levels (Fig. 4C–E), while NNMT knockdown reduced these levels (Fig. 4C–E). Next, we explored whether NNMT promoted the proliferation, migration and invasion of iCCA through the Warburg effect by adding 4 μM 2-DG, a glycolysis inhibitor, to the culture medium and found that 2-DG inhibited the progression of iCCA cells and reversed the proliferative and metastasis premetastatic effects of NNMT overexpression (Fig. 4F–H). Since NNMT can indirectly affect intracellular NAD+ levels through NMN, this may affect cellular metabolism and proliferation. Therefore, we added NMN to the culture medium to test its effect on the proliferation and metastasis of iCCA cells. The results showed that the addition of NMN did not significantly affect the proliferation and metastasis of iCCA cells (Supplementary Fig. 2).

### NNMT promotes the aerobic glycolysis process in iCCA by increasing EGFR expression

**Fig. 5:**
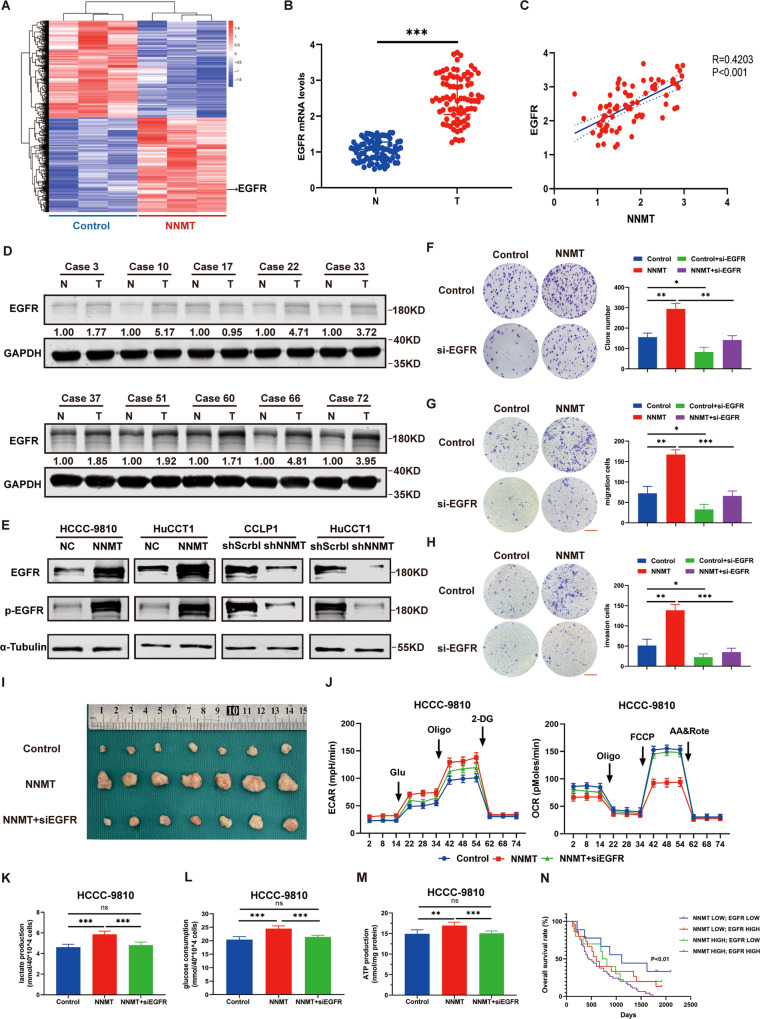
NNMT regulates EGFR expression and the Warburg effect in iCCA cells. **A** Heat map of differentially expressed genes in HCCC-9810-Control and HCCC-9810-NNMT cells. **B** The expression level of the EGFR mRNA in iCCA and adjacent tissues. **C** The mRNA expression level was used to determine the correlation between NNMT and EGFR expression. **D** The expression level of the EGFR protein in iCCA and adjacent tissues. **E** WB was used to detect the expression levels of the EGFR and p-EGFR protein in iCCA cells transfected with NNMT lentivirus. **F** Colony formation assays detected the proliferation of iCCA cells transfected with si-EGFR. **G** Transwell assays detected the migration of iCCA cells transfected with si-EGFR. **H** Transwell assays detected the invasion of iCCA cells transfected with si-EGFR. Scale bars: 100 μm. **I** In vivo experiments were conducted to detect the effect of si-EGFR on tumor proliferation in nude mice. **J** The Seahorse X96 analyzer detects the acid excretion capacity and oxygen consumption capacity of iCCA cell lines. **K**–**M** lactic acid and ATP production and glucose consumption in iCCA cells transfected with si-EGFR. **N** The Kaplan–Meier method was used to determine the overall survival rate of patients with iCCA (data are mean ± SEM, *P < 0.05, **P < 0.01, ***P < 0.001, *n* = 3).

We performed NNMT transcriptome sequencing to further clarify the molecular mechanism by which NNMT promotes the Warburg effect in iCCA cells, and the results are shown in Fig. 5A. Among the many differentially expressed genes, we focused on the EGFR gene, because compared with the Control group, the EGFR expression in the NNMT-OE group changed significantly and EGFR has been reported to be closely related to the occurrence and development of iCCA and the Warburg effect [23, 24]. Therefore, we speculated that NNMT may exert its biological effects by regulating EGFR expression. We verified this hypothesis by detecting EGFR mRNA and protein levels in iCCA tissues and found that EGFR was expressed at high levels in iCCA tissues (Fig. 5B, D). NNMT and EGFR expression levels were positively correlated in iCCA samples (Fig. 5C). Subsequently, we found that NNMT increased EGFR expression and p-EGFR levels in iCCA cells (Fig. 5E). Therefore, we postulated that NNMT regulates EGFR expression in iCCA cells.

Next, we transfected si-EGFR and its control sequence in the HCCC-9810. Based on the results of colony formation assays and Transwell experiments, si-EGFR reversed the increases in proliferation, migration, and invasion induced by NNMT overexpression (Fig. 5F–H). We performed the same process with the p-EGFR inhibitor erlotinib, and a similar result was obtained as with si-EGFR (Supplementary Fig. 3A–C). In vivo experimental results showed that inhibiting EGFR expression reduced the volume of subcutaneous tumors (Fig. 5I). Next, we measured the OCR and ECAR and found that the transfection of si-EGFR reversed the decrease in OCR and increase in ECAR caused by NNMT overexpression (Fig. 5J). The transfection of si-EGFR or treatment with erlotinib inhibited ATP and lactate production and glucose consumption (Fig. 5K–M and Supplementary Fig. 3D–F). Therefore, we propose that NNMT regulates the Warburg effect by inducing EGFR expression to promote iCCA proliferation and metastasis. Then, we grouped the clinical samples according to the expression of NNMT and EGFR and analyzed the survival curve. Patients with high expression of NNMT and EGFR had a shorter overall survival time (Fig. 5N).

### NNMT induces EGFR expression by inhibiting the methylation of H3K9me3 and H3K27me3

**Fig. 6:**
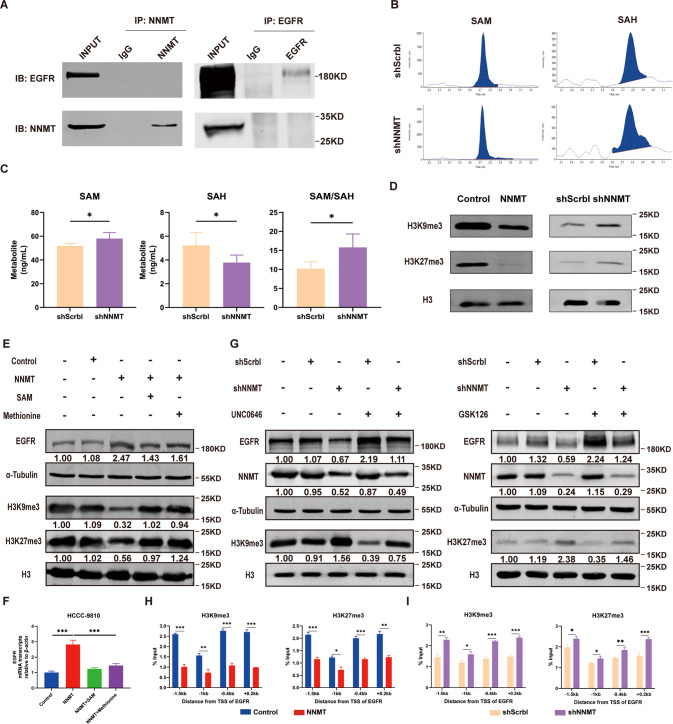
NNMT induces EGFR expression by decreasing the levels of H3K9me3 and H3K27me3. **A** Co-IP analysis of the binding between NNMT and EGFR in HCCC-9810 cells transfected with the Flag-NNMT lentivirus (left panel). Co-IP analysis of the binding between NNMT and EGFR in HCCC-9810 cells transfected with the EGFR plasmid (right panel). **B**, **C** HPLC detected the levels of SAM and SAH and the ratio of SAM/SAH in iCCA cells. **D** WB analysis of the effect of NNMT on the levels of H3K9me3 and H3K27me3 in iCCA cells. **E**, **F** The effect of SAM (500 μM) or methionine (100 μM) on the methylation level and EGFR expression in iCCA cells. **G** WB detection and analysis of the EGFR protein level in cells treated with 5 μM UNC0646 or 5 μM GSK126 in the culture medium. **H**, **I** ChIP analyses of the degree of enrichment of H3K9me3 (left panel) or H3K27me3 (right panel) at the EGFR promoter region (data are mean ± SEM, *P < 0.05, **P < 0.01, ***P < 0.001, *n* = 3).

We conducted an immunoprecipitation experiment to explore the mechanism by which NNMT promotes EGFR expression. Unfortunately, we did not observe binding between the two proteins (Fig. 6A). Although NNMT is involved in the methylation metabolism process, it converts the universal methyl donor SAM into SAH, which in turn affects the level of histone methylation in the cell [25, 26]. Therefore, we speculated that NNMT may affect EGFR expression by modulating the level of histone methylation in iCCA cells. Next, we tested the levels of SAM and SAH in the cells. The intracellular SAM level increased, the SAH level decreased, and the SAM/SAH ratio increased after NNMT knockdown (Fig. 6B, C). Overexpression of NNMT reduced the levels of H3K9me3 and H3K27me3 in iCCA cells, while knockdown of NNMT increased these levels (Fig. 6D). The addition of the universal methyl donor SAM or methionine to the medium rescued the intracellular methylation level and inhibited EGFR expression (Fig. 6E, F). Therefore, we suggest that NNMT reduces H3K9me3 and H3K27me3 levels by consuming methyl donors. The addition of UNC0646 (H3K9me3 methyltransferase inhibitor) or GSK126 (H3K27me3 methyltransferase inhibitor) to the culture medium increased the expression of the EGFR mRNA and protein (Fig. 6G and Supplementary Fig. 4A, B). ChIP assays further revealed that NNMT overexpression reduced the enrichment of H3K9me3 and H3K27me3 at the EGFR promoter region compared with the control group (Fig. 6H, I).

### NNMT promotes the Warburg effect by activating the EGFR-STAT3 pathway

**Fig. 7:**
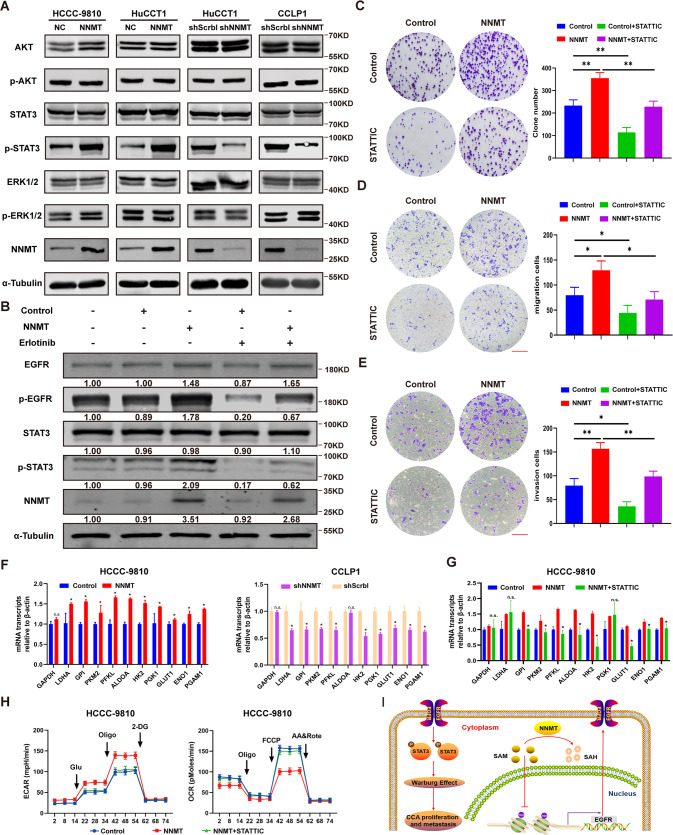
NNMT regulates the Warburg effect in iCCA through the EGFR-STAT3 axis. **A** The expression level of EGFR-related signaling proteins in the cells was measured using western blotting. **B** After treatment with 10 μM erlotinib, the levels of EGFR, p-EGFR, and p-STAT3 in the cells were determined using western blotting. **C** Colony formation assay using the cell line treated with 5 μM STATTIC. **D** Migration test after treatment of the cell line with 5 μM STATTIC. **E** Invasion test after treatment of the cell line with 5 μM STATTIC. Scale bars: 100 μm. **F** The effect of NNMT on the mRNA levels of the key enzymes involved in glycolysis. **G** The effect of 5 μM STATTIC on the mRNA levels of the key enzymes involved in glycolysis in cell lines overexpressing NNMT. **H** The Seahorse X96 analyzer detects the ability of iCCA cell lines to excrete acid and the oxygen consumption rate after treatment with 5 μM STATTIC. **I** Schematic presentation of the pathway by which NNMT facilitated iCCA proliferation and metastasis (data are mean ± SEM, *P < 0.05, **P < 0.01, ***P < 0.001, *n* = 3).

EGFR activates a variety of effector pathways in cells, and thus we wanted to explore which effector pathway NNMT-EGFR activates to exert its cancer-promoting effect. Therefore, the signaling pathways were investigated using western blotting to evaluate the levels of phosphorylated AKT, STAT3, and ERK. The level of p-STAT3 was significantly increased after NNMT overexpression, and NNMT knockdown decreased STAT3 phosphorylation (Fig. 7A). The addition of erlotinib or si-EGFR to the medium reduced the level of p-STAT3 (Fig. 7B and Supplementary Fig. 4C); therefore, we conclude that NNMT promotes tumor proliferation and metastasis through the EGFR-STAT3 pathway. We then determined whether the STAT3 signaling pathway was required for NNMT-mediated iCCA progression. The STAT3 phosphatase inhibitor STATTIC inhibited the cancer-promoting effect of NNMT overexpression on colony formation and migration in transwell experiments (Fig. 7C–E). Next, we performed qRT–PCR to determine the expression of key enzymes in the glycolysis pathway. NNMT overexpression generally promoted an increase in the expression of key enzymes in the glycolysis pathway, and NNMT knockdown reduced the levels of these enzymes to varying degrees (Fig. 7F). Interestingly, STATTIC also inhibited the expression of these key enzymes to varying degrees (Fig. 7G). The reduction in the p-STAT3 level also inhibited the aerobic glycolysis pathway (Fig. 7H and Supplementary Fig. 4D–F). In summary, NNMT induces EGFR expression by reducing the levels of H3K9me3 and H3K27me3 in the EGFR promoter region, thereby activating the EGFR-STAT3 signaling pathway to promote the intracellular glycolysis process, proliferation, and metastasis in iCCA cells (Fig. 7I).

## Discussion

Intrahepatic cholangiocarcinoma (iCCA) is the second most common primary malignant tumor in the liver. Because of its aggressiveness, poor prognosis, and the limited treatment strategies for malignant tumors, most patients with iCCA survive less than one year after diagnosis [1, 4]. Therefore, the identification of effective treatments and molecular targets for iCCA has become an urgent task to be solved. NNMT is a cytoplasmic enzyme that belongs to the N-methyltransferase family [5]. NNMT participates in the regulation of various metabolic pathways and is involved in tumor progression and metastasis [20, 27]. However, its role in iCCA has not been explored. In this study, we found that NNMT was significantly upregulated in iCCA tissue samples compared with adjacent noncancerous tissues. Furthermore, the expression of NNMT was related to the tumor TNM stage and a poor prognosis in iCCA tissues. In vivo and in vitro experiments also indicated that NNMT increased the proliferation and metastasis of iCCA cells. Thus, NNMT plays an important role in promoting tumor growth during the progression of iCCA.

Tumor cells often exhibit increased aerobic glycolysis, also known as the Warburg effect. This effect is presumed to more efficiently provide energy for tumor cells and modulate the acidic environment to increase tumor cell growth, metastasis ability, and chemotherapy resistance [28]. A large number of studies have reported that NNMT is associated with cell metabolism, including lipid metabolism, methyl metabolism, and glucose metabolism [26, 27, 29–31]. These observations prompted us to speculate whether NNMT is related to the Warburg effect. As expected, we confirmed that NNMT overexpression increases the ECAR and lactate production, while NNMT knockdown reduces the aerobic glycolytic phenotype of iCCA cell lines. In addition, NNMT indirectly regulates the expression of multiple glycolysis-related genes in the glycolysis pathway [32].

In recent years, many studies have shown that EGFR gene expression is increased in CCA cells, which is associated with the malignant biological behavior of tumors [33–36]. The EGFR inhibitor erlotinib has been used as a monotherapy for patients with non-small cell lung cancer (NSCLC) and combined with gemcitabine for patients with pancreatic cancer to prolong their survival [37, 38]. Furthermore, based on the RNA-seq results, NNMT induces the expression of the EGFR transcript. Therefore, in this study, we regarded EGFR as a downstream target of NNMT. We also confirmed that NNMT exerts a positive regulatory effect on the EGFR-STAT3 axis, and the EGFR inhibitor erlotinib inhibits the malignant biological characteristics of iCCA caused by high NNMT expression. Accordingly, we reasonably speculate that NNMT-EGFR has a high possibility of serving as a molecular target for the treatment of iCCA and has strong clinical transformation value.

Histone methylation is a reversible epigenetic modification that plays an important role in biological processes [33]. NNMT is a methyltransferase that participates in histone methylation by regulating SAM methylation metabolism [39–41]. In this article, we used HPLC and western blotting to detect the SAM/SAH ratio and the methylation levels of H3K9me3 and H3K27me3, respectively. NNMT regulates the expression of EGFR by consuming SAM methyl donors. However, we did not determine whether this regulatory mechanism is the main pathway by which NNMT regulates downstream gene expression. Therefore, further explorations of the molecular mechanism of NNMT are required.

In summary, we have presented evidence showing that the EGFR-STAT3 pathway is the main downstream effector of NNMT in iCCA, and NNMT activates the EGFR–STAT3 signaling axis to regulate glycolysis in iCCA cells by reducing the levels of H3K9me3 and H3K27me3 at the EGFR promoter region, which is a unique mechanism to promote the proliferation of iCCA cells. Our findings provide confident support for the important role of NNMT as a prognostic indicator and therapeutic target for iCCA.

## Supplementary information

Supplementary table 1-4

Supplementary figure 1-4

Supplementary methods

## Data Availability

Data, materials, and software information supporting the conclusions of this article are included within the article and its Supplementary materials.
