# Supplementary material for: NNMT promotes the progression of intrahepatic cholangiocarcinoma by regulating aerobic glycolysis via the EGFR-STAT3 axis
Source: Oncogenesis. 2022 Jul 18;11(1):39. doi: 10.1038/s41389-022-00415-5 (PMC9293979; doi:10.1038/s41389-022-00415-5)
Supplement: Supplementary file 1 — Supplementary table 1-4 [file 41389_2022_415_MOESM1_ESM.docx]

**Supplementary table 1 Clinic characteristics.**

| Features | NNMT staining | | P value |
| --- | --- | --- | --- |
|  | Positive(n=55)  n (%) | Low (n=25)  n (%) |  |
| Age(years) |  |  |  |
| ≥60 | 30(54.55%) | 12(48%) | 0.6349 |
| <60 | 25(45.45%) | 13(52%) |  |
| Gender |  |  |  |
| Male | 29(52.73%) | 14(56%) | 0.8136 |
| Female | 26(47.27%) | 11(44%) |  |
| Histological differentiation | |  |  |
| Well | 10(18.18%) | 5(20%) | 0.8623 |
| Moderate | 32(58.18%) | 12(48%) |  |
| Poor | 13(23.64%) | 8(32%) |  |
| TNM stage |  |  |  |
| Ⅰ | 0(0) | 3(12%) | 0.0015 |
| Ⅱ | 17(30.91%) | 15(60%) |  |
| Ⅲ | 29(52.73%) | 5(20%) |  |
| Ⅳ | 9(16.36%) | 2(8%) |  |
| Lymph node metastasis |  |  |  |
| No | 32(58.18%) | 18(72%) | 0.0379 |
| Yes | 23(41.82%) | 7(28%) |  |

**Supplementary table 2 Primer information**

| Gene Name | Sequence (5′-3′) |
| --- | --- |
| NNMT | F: AGCTGGAGAAGTGGCTGAAG |
|  | R: TGGACCCTTGACTCTGTTCC |
| EGFR | F: CCCACTCATGCTCTACAACCC |
|  | R: TCGCACTTCTTACACTTGCGG |
| PFKL | F: GCTGGGCGGCACTATCATT |
|  | R: TCAGGTGCGAGTAGGTCCG |
| HK2 | F: TTGACCAGGAGATTGACATGGG |
|  | R: CAACCGCATCAGGACCTCA |
| GLUT1 | F: ATTGGCTCCGGTATCGTCAAC |
|  | R: GCTCAGATAGGACATCCAGGGTA |
| PGAM1 | F: GTGCAGAAGAGAGCGATCCG |
|  | R: CGGTTAGACCCCCATAGTGC |
| PGK1 | F: GACCTAATGTCCAAAGCTGAGAA |
|  | R: CAGCAGGTATGCCAGAAGCC |
| ENO1 | F: GCCGTGAACGAGAAGTCCTG |
|  | R: ACGCCTGAAGAGACTCGGT |
| LDHA | F: TTGACCTACGTGGCTTGGAAG |
|  | R: GGTAACGGAATCGGGCTGAAT |
| GPI | F: CAAGGACCGCTTCAACCACTT |
|  | R: CCAGGATGGGTGTGTTTGACC |
| PKM2 | F: ATGTCGAAGCCCCATAGTGAA |
|  | R: TGGGTGGTGAATCAATGTCCA |
| ALDOA | F: AACTTTCCTCTGCCTAGCCC |
|  | R: GTACAGGCACAGTCGCAGAG |
| GAPDH | F: CATGAGAAGTATGACAACAGCCT |
|  | R: AGTCCTTCCACGATACCAAAGT |
| beta-ACTIN | F: CCAAGGCCAACCGCGAGAAGATGAC |
|  | R: AGGGTACATGGTGGTGCCGCCAG |
| EGFR-promoter-ChIP-1 | F: GCTGTCTTTTTGGGCACTCA |
|  | R: TGGGAAGCACAACAGTGGAA |
| EGFR-promoter-ChIP-2 | F: GGTTGCTCCCCTTCAGAGAC |
|  | R: CCCCTCCTCTCTTCACGAGA |
| EGFR-promoter-ChIP-3 | F: CATTATCCGACGCTGGCTCT |
|  | R: CCAGGTCGAGCCAAATCTGT |
| EGFR-promoter-ChIP-4 | F: CCCCTGACTCCGTCCAGTAT |
|  | R: TTCTTTTCCTCCAGAGCCCG |

**Supplementary table 3 Antibody information**

| Antibody | Host | Product number | Company | Dilution |
| --- | --- | --- | --- | --- |
| NNMT | Rabbit | OAAN01939 | Aviva System Biology | 1:1000(WB)  1:200(IHC)  1:50(IP) |
| NNMT | Mouse | ab119758 | Abcam | 1:150(IF) |
| EGFR | Rabbit | #4267 | Cell Signaling Technology | 1:2000(WB)  1:100(IP) |
| p-EGFR | Rabbit | #3777 | Cell Signaling Technology | 1:1000(WB) |
| STAT3 | Rabbit | #4904 | Cell Signaling Technology | 1:1000(WB) |
| p-STAT3 | Rabbit | #9145 | Cell Signaling Technology | 1:1000(WB) |
| AKT | Rabbit | #4691 | Cell Signaling Technology | 1:1000(WB) |
| p-AKT | Rabbit | #4060 | Cell Signaling Technology | 1:1000(WB) |
| p44/42 MAPK | Rabbit | #4695 | Cell Signaling Technology | 1:1000(WB) |
| p-p44/42 MAPK | Rabbit | #4370 | Cell Signaling Technology | 1:1000(WB) |
| H3 | Rabbit | #4499 | Cell Signaling Technology | 1:2000(WB) |
| H3K9me3 | Rabbit | #13969 | Cell Signaling Technology | 1:1000(WB) |
| H3K27me3 | Rabbit | #9733 | Cell Signaling Technology | 1:1000(WB) |
| FLAG | Rabbit | #14973 | Cell Signaling Technology | 1:1000(WB)  1:50(IP) |
| CK-19 | Rabbit | 10712-1-AP | Proteintech | 1:200(IF) |
| Ki-67 | Mouse | #9449 | Cell Signaling Technology | 1:500(IHC) |
| GAPDH | Mouse | 60004 | Proteintech | 1:10000(WB) |
| α-Tubulin | Mouse | 66031 | Proteintech | 1:10000(WB) |

**Supplementary table 4 NNMT immunohistochemical score of iCCA patients**

| Number | NNMT score | NNMT group |
| --- | --- | --- |
| 1 | 1 | LOW |
| 2 | 8 | HIGH |
| 3 | 9 | HIGH |
| 4 | 12 | HIGH |
| 5 | 4 | LOW |
| 6 | 1 | LOW |
| 7 | 12 | HIGH |
| 8 | 12 | HIGH |
| 9 | 9 | HIGH |
| 10 | 0 | LOW |
| 11 | 12 | HIGH |
| 12 | 1 | LOW |
| 13 | 9 | HIGH |
| 14 | 3 | LOW |
| 15 | 12 | HIGH |
| 16 | 8 | HIGH |
| 17 | 6 | HIGH |
| 18 | 6 | HIGH |
| 19 | 0 | LOW |
| 20 | 12 | HIGH |
| 21 | 9 | HIGH |
| 22 | 1 | LOW |
| 23 | 9 | HIGH |
| 24 | 1 | LOW |
| 25 | 9 | HIGH |
| 26 | 9 | HIGH |
| 27 | 9 | HIGH |
| 28 | 6 | HIGH |
| 29 | 9 | HIGH |
| 30 | 2 | LOW |
| 31 | 12 | HIGH |
| 32 | 6 | HIGH |
| 33 | 9 | HIGH |
| 34 | 8 | HIGH |
| 35 | 6 | HIGH |
| 36 | 12 | HIGH |
| 37 | 6 | HIGH |
| 38 | 6 | HIGH |
| 39 | 12 | HIGH |
| 40 | 0 | LOW |
| 41 | 4 | LOW |
| 42 | 9 | HIGH |
| 43 | 8 | HIGH |
| 44 | 2 | LOW |
| 45 | 2 | LOW |
| 46 | 9 | HIGH |
| 47 | 2 | LOW |
| 48 | 9 | HIGH |
| 49 | 4 | LOW |
| 50 | 8 | HIGH |
| 51 | 8 | HIGH |
| 52 | 9 | HIGH |
| 53 | 9 | HIGH |
| 54 | 6 | HIGH |
| 55 | 3 | LOW |
| 56 | 12 | HIGH |
| 57 | 8 | HIGH |
| 58 | 9 | HIGH |
| 59 | 9 | HIGH |
| 60 | 4 | LOW |
| 61 | 9 | HIGH |
| 62 | 4 | LOW |
| 63 | 1 | LOW |
| 64 | 9 | HIGH |
| 65 | 4 | LOW |
| 66 | 6 | HIGH |
| 67 | 3 | LOW |
| 68 | 4 | LOW |
| 69 | 6 | HIGH |
| 70 | 6 | HIGH |
| 71 | 9 | HIGH |
| 72 | 3 | LOW |
| 73 | 1 | LOW |
| 74 | 8 | HIGH |
| 75 | 6 | HIGH |
| 76 | 8 | HIGH |
| 77 | 9 | HIGH |
| 78 | 9 | HIGH |
| 79 | 6 | HIGH |
| 80 | 6 | HIGH |
