# Supplementary material for: NNMT promotes the progression of intrahepatic cholangiocarcinoma by regulating aerobic glycolysis via the EGFR-STAT3 axis
Source: Oncogenesis. 2022 Jul 18;11(1):39. doi: 10.1038/s41389-022-00415-5 (PMC9293979; doi:10.1038/s41389-022-00415-5)
Supplement: Supplementary file 2 — Supplementary figure 1-4 [file 41389_2022_415_MOESM2_ESM.pdf]

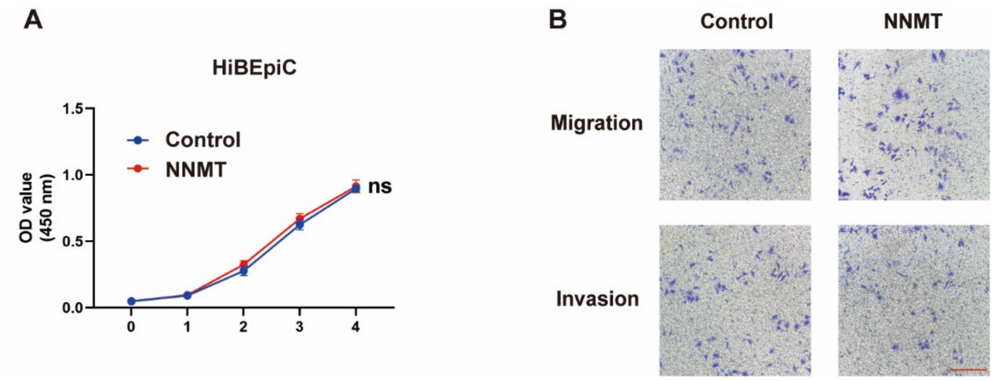

Supplementary Figure 1

A The CCK-8 experiment was used to evaluate the proliferation of NNMT-overexpressing and the control HiBEpiC cell line. **B** Transwell experiments verified the migration and invasion of HiBEpiC cells overexpressing NNMT, as well as the control HiBEpiC cells. Scale bars: 100 μm. (data are mean ± SEM, *P < 0.05, **P < 0.01, ***P<0.001, n = 3)

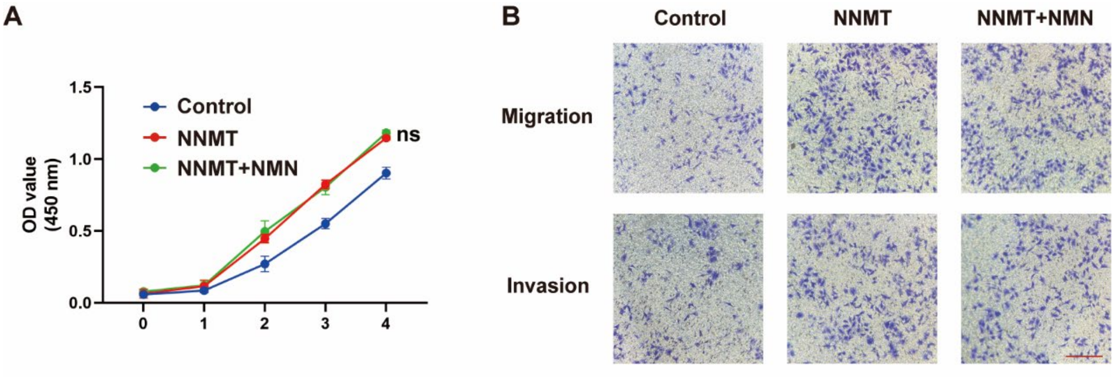

Supplementary Figure 2

A CCK-8 assay detecting cell proliferation after the addition of 1 mM NMN to the culture medium. **B** The Transwell experiment detected cell migration and invasion after the addition of 1 mM NMN to the culture medium. Scale bars: 100 μm. (data are mean ± SEM, *P < 0.05, **P < 0.01, ***P<0.001, n = 3)

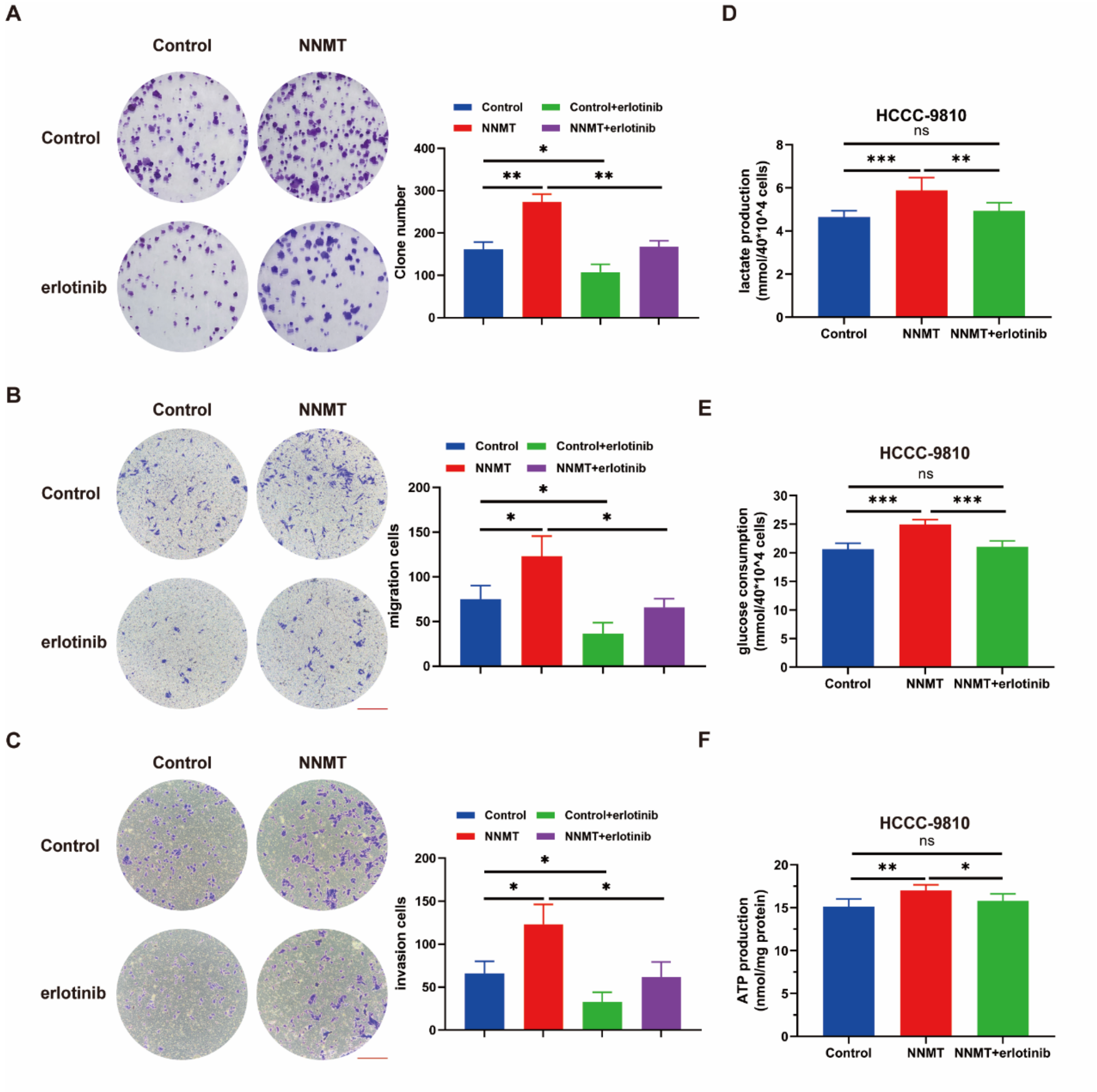

Supplementary Figure 3

A Colony formation assay detecting cell proliferation after the addition of 10 μM erlotinib to the culture medium. **B-C** The Transwell experiment detected cell migration (B) and invasion (C) after the addition of 10 μM erlotinib to the culture medium. Scale bars: 100 μm. **D** Lactic acid production was inhibited by the 10 μM erlotinib treatment. **E** Glucose consumption was inhibited by the 10 μM erlotinib treatment. **F** ATP production was inhibited by the 10 μM erlotinib treatment. (data are mean ± SEM, *P < 0.05, **P < 0.01, ***P<0.001, n = 3)

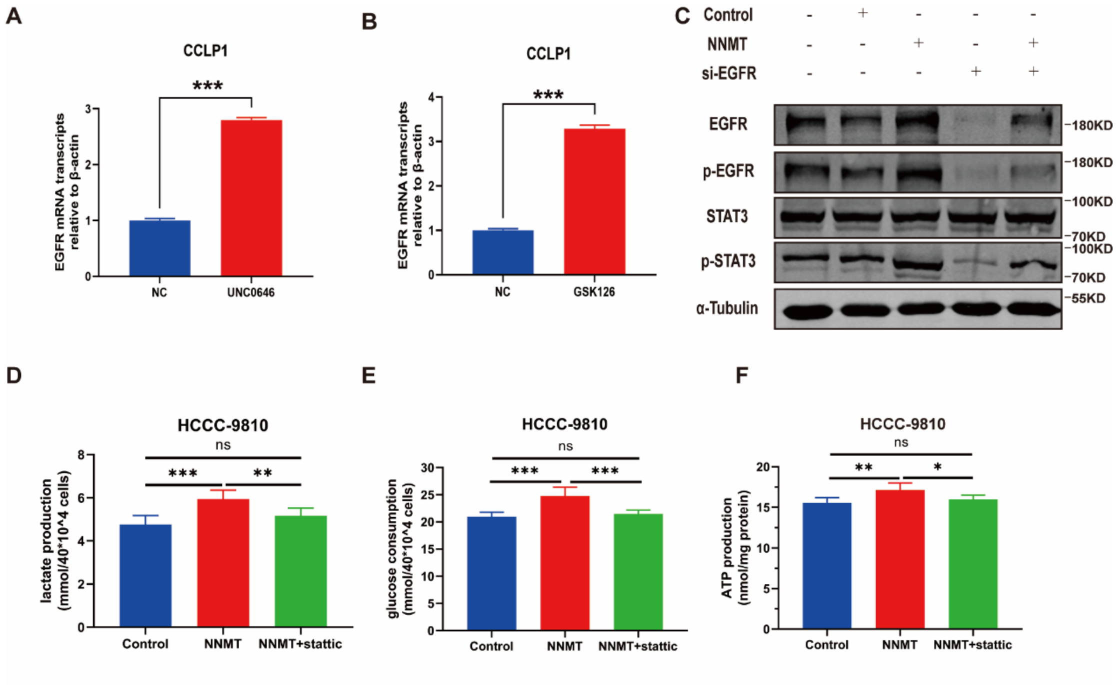

Supplementary Figure 4

A-B Changes in EGFR mRNA levels were assessed after the addition of 5 μM UNC0646 (A) or 5 μM GSK126 (B) to the culture medium. **C** After the transfection of si-EGFR, the levels of EGFR, p-EGFR, STAT3 and p-STAT3 in the cells were determined using western blotting. **D** Lactic acid production was inhibited by the 5 μM STATTIC treatment. **E** Glucose consumption was inhibited by the 5 μM STATTIC treatment. **F** ATP production was inhibited by the 5 μM STATTIC treatment. (data are mean ± SEM, *P < 0.05, **P < 0.01, ***P<0.001, n = 3)
