# Supplementary material for: NNMT promotes the progression of intrahepatic cholangiocarcinoma by regulating aerobic glycolysis via the EGFR-STAT3 axis
Source: Oncogenesis. 2022 Jul 18;11(1):39. doi: 10.1038/s41389-022-00415-5 (PMC9293979; doi:10.1038/s41389-022-00415-5)
Supplement: Supplementary file 3 — Supplementary methods [file 41389_2022_415_MOESM3_ESM.pdf]

Supplementary Methods

Cell lines

The HuCCT1 cell line was kindly donated by the Cancer Cell Repository, Tohoku University, Japan. HCCC-9810, RBE, QBC-939 and CCLP1 cells were purchased from Shanghai Bioleaf Biotech Co., Ltd (Shanghai, China). The normal human intrahepatic biliary cell line (HIBEpiC) was purchased from ScienCell Research Laboratories (Carlsbad, CA). CCA cell lines were cultured in RPMI 1640 (Gibco™, Thermo Fisher Scientific, Waltham, MA, USA) supplemented with 10% fetal bovine serum (FBS; Gibco™, Thermo Fisher Scientific, Waltham, MA, USA) and 1% penicillin-streptomycin (Gibco™, Thermo Fisher Scientific, Waltham, MA, USA).

Western blotting

Tissues and cells were lysed with RIPA buffer, and proteins were harvested. Forty micrograms of protein were separated on SDS–PAGE gels and transferred to PVDF membranes (Invitrogen). Detailed descriptions of the antibodies are provided in Additional file 2. Proteins were visualized and quantified using an Odyssey CLx Imaging System (LI-COR Biosciences, USA).

Immunohistochemistry (IHC)

IHC staining was performed as reported in our previous study [22]. Briefly, tissue sections were deparaffinized, rehydrated, and then blocked with 10% normal goat serum. An anti-NNMT antibody (Aviva Systems Biology, San Diego, CA) (1:200 dilution) or an anti-Ki-67 antibody (Cell Signaling Technology, Danvers, MA) (1:500 dilution) was incubated with the sections overnight at 4 °C. Sections were then sequentially incubated with a secondary antibody (Vector lab, Burlingame, CA) for 1 h at room temperature and Vectastain Elite ABC reagent (Vector lab) for 30 min. Tissue sections were then stained with diaminobenzidine (DAB kit; Vector lab) and counterstained with hematoxylin (Sigma). The density

of IHC staining was analyzed using Image-Pro Plus v6.2 software, and the median density values of all the slides with positive staining were used as the cutoff to define high or low expression subgroups. NNMT staining intensity was scored as 0 (negative), 1 (weak), 2 (moderate) and 3 (strong). The staining extent was scored based on the percentage of positive cells using the following scale: 0 (negative), 1 (0.01–25%), 2 (25.01–50%), 3 (50.01–75%), and 4 (75.01–100%). The histologic score for each section was calculated with the following formula: histologic score = proportion score × intensity score. Thus, the total score could be 0, 1, 2, 3, 4, 6, 8, 9, or 12, and the staining could be classified as negative/low (0, 1, 2, 3, 4) or positive/high (6, 8, 9, 12). (The detailed scores of each sample are shown in Supplementary Table 4)

Immunofluorescence staining

Tissue sections were deparaffinized, rehydrated, and then blocked with 10% normal goat serum. An anti-NNMT antibody (Abcam, ab119758) (1:150 dilution) and an anti-CK-19 antibody (Proteintech, 10712-1-AP) (1:200 dilution) was incubated with the sections overnight at 4 °C. The following day, cells were incubated with a fluorescent secondary antibody for 1 h. Finally, nuclei were counterstained with DAPI, and the images were photographed under a confocal laser scanning microscope.

RNA isolation and qPCR assay

Total RNA was extracted from cells and tissues using an RNA Miniprep Kit (Axygen), quantified with a Nanodrop ND-2000 spectrophotometer and reverse transcribed into cDNAs using a High Capacity Reverse Transcription Kit (Applied Biosystems). Real-time PCR was performed using Power SYBR Green PCR Master Mix (Applied Biosystems) and an ABIPRISM 7500HT instrument (Applied Biosystems). The expression levels of the indicated mRNAs were determined using the ΔΔCt method with beta-actin as an internal control. A complete list of primer sets is provided in Additional file 2.

Cell transfection

Lenti-shNNMT and the corresponding control vectors were designed and purchased from GeneChem (Shanghai, China). Lenti-NNMT and the corresponding control vectors were designed and purchased from HanBio (Shanghai, China). Following transfection, successfully transfected cells were selected in the presence of puromycin (Sigma–Aldrich Corp., St. Louis, MO, USA) for 2 weeks. The sequence of the shRNA was 5’-CCGGGTGACCTATGTGTGTGATCTTCTCGAGAAGATCACACACATAGGTCACTTTTTG-3’. For siRNA transfection, Lipofectamine 2000 reagent was used according to the manufacturer’s protocol, and the siRNA sequence was 5’-GCUAUGAGAUGGAGGAAGA-3’.

CCK-8 assay

For this experiment, 500-1000 cells were seeded in each well of a 96-well plate. The optical density (OD) value was determined by adding the CCK-8 (Dojindo, Japan) solution at the indicated time points and incubating the cells for two hours at 37 °C.

Colony formation assays

For colony formation assays, 500-1000 cells were seeded into each well of 6-well plates and cultured for 14 days. Then, the colonies were fixed with 4% paraformaldehyde (PFA) and stained with 0.5% crystal violet to visualize colonies.

EdU assay

An EdU analysis kit (Beyotime, China) was used to detect DNA synthesis and cell proliferation. A total of 100,000 treated CCA cells were seeded in each well of a 24-well plate and cultured overnight. The next day, an EdU solution (10 μM) was added to each well of the 24-well plate and incubated for 2 hours. Next, 4% formalin was applied, and the CCA cells were fixed at room temperature for 2 hours. In

the next step, CCA cells were infiltrated with 0.5% Triton X-100 for 10 minutes, then Click Additive Solution (200 μL) was added to stain EdU for 30 minutes, and Hoechst 33342 (200 μL) was added to stain the nucleus. Finally, a Nikon microscope (Nikon Japan) was used to observe DNA synthesis and cell proliferation, which were reflected by the red and blue signals, respectively.

Wound-healing, migration and invasion assays

For the wound-healing assay, 5-8×10^5 CCA cells were cultured overnight and allowed to grow to confluence. Cells were gently scratched with a sterile 1000 μL pipette tip, washed three times with PBS and then incubated with medium containing 0.5% FBS. Images were captured at 0 h, 12 h or 24 h using a Nikon Eclipse TS100 microscope.

Determination of SAM and SAH levels

Concentrations of SAM and SAH were determined using reversed-phase high performance liquid chromatography (HPLC). The cells (1x107) were centrifuged, washed twice with PBS, and maintained on ice. The cell pellets were subsequently homogenized in 100 μL of 0.4 M ice-cold perchloric acid. Homogenates were centrifuged at 15,000 g for 15 min at 4 °C. The supernatants were collected and stored at -80 °C until analysis. The supernatant of each sample was loaded on a C18 column (SunFire, Ireland) and analyzed using a Waters HPLC system (Milford, MA). The two mobile phases were as follows: mobile phase A consisted of 50 mM NaH2PO4 and 8 mM heptanesulfonic acid (pH=3.0), and mobile phase B contained methanol. Isocratic elution was achieved at a flow rate of 1 mL/min with the following parameters: 80% mobile phase A and 20% mobile phase B. The total run time was 15 min, and the injection volume was 20 μL. The absorbance of analytes was recorded by a UV detector at 254 nm. The SAM and SAH standards purchased from Sigma-Aldrich (St. Louis, MO, USA) were used to identify the elution peaks, and the cellular SAM and SAH levels were calculated using automatic peak area

integration.

Coimmunoprecipitation assay

Total proteins were extracted with cell lysis buffer (Cell Signaling Technology, Danvers, MA) supplemented with protease inhibitor and phosphatase inhibitor (Cell Signaling Technology, Danvers, MA). The lysate was incubated with anti-NNMT (Aviva), anti-EGFR (Cell Signaling Technology, Danvers, MA) and IgG antibodies (Cell Signaling Technology, Danvers, MA) (as a negative control) at 4 °C overnight with gentle rotation. Then, the protein-antibody complexes were incubated with Protein A/G Plus Agarose (Santa Cruz, USA) for 5 h at 4 °C with gentle rotation. Immunoprecipitates were then collected by centrifugation at 14000×g for 30 s at 4 °C, after which the bead complexes were washed three times with cell lysis buffer. After the final wash, proteins were eluted from Protein A/G agarose by boiling in 5X SDS sample buffer at 100 °C for 5 mins before western blotting analyses.

Animal studies

Male BALB/c nude mice (4–5 weeks old) were obtained from Beijing Vital River Laboratory Animal Technology Co., Ltd. and housed in a specific pathogen-free facility. All animal experiments were conducted in accordance with the standard protocols of the Institutional Animal Care and Use Committee of Harbin Medical University. For the analysis of xenograft tumor growth, 3×10^6 CCA cells suspended in 200 μL of PBS were injected into the flanks of mice (n = 7/group). Tumor size was measured weekly with Vernier calipers, and the tumor volume was calculated using the formula: volume = length × $(\text{width})^2$ × 0.5. The mice were monitored for tumor formation over 4 weeks. Long-distance lung metastasis was evaluated by first transfecting cells with a lentivirus containing firefly luciferase, and then 3×10^6 CCA cells suspended in 150 μL of PBS were injected into nude mice (n = 7/group) through the tail vein. D-Luciferin (Gold Biotechnology, Hopkinton, MA) was administered

intraperitoneally at 100 mg/kg body weight, and bioluminescence was detected with a Berthold NIGHTOWL LB983 imaging machine. Mice were euthanized at 12 weeks, and lung metastases were confirmed by H&E staining. Peritoneal metastasis was evaluated by injecting 3×10^6 CCA cells in 200 μL of PBS into the intraperitoneal cavity of 4–5-week-old male BALB/c mice (n = 7/group). Mice were imaged at 4 weeks after the injection and then euthanized, and the number of metastatic nodules was counted.
